# As Time Goes By: Event File Decay Does Not Unleash Inhibition of Return

**DOI:** 10.5334/joc.422

**Published:** 2025-01-15

**Authors:** Lars-Michael Schöpper, Christian Frings

**Affiliations:** 1 University of Trier, Department of Cognitive Psychology, Germany; 2 Institute for Cognitive and Affective Neuroscience (ICAN), University of Trier, Germany

**Keywords:** Action control, attentional orienting, response-stimulus-interval (RSI), inhibition of return (IOR), binding and retrieval

## Abstract

Inhibition of return (IOR) refers to a location repetition cost typically observed when signaling the detection of or localizing sequentially presented stimuli repeating or changing their location. In discrimination tasks, however, IOR is often reduced or even absent; here, effects of binding and retrieval are thought to take place. Information is bound into an event file, which upon feature repetition causes retrieval, leading to partial repetition costs. It is assumed that the presence of retrieval-based effects masks the observation of IOR. Yet, some evidence suggests that long intervals between stimuli can lead to IOR in tasks in which usually mostly binding effects are observed. We hypothesized that with an increasing interval between prime response and probe onset (response stimulus interval, RSI), event files will decay and decreasingly mask IOR. In turn, IOR should be strongest at longest intervals. In the current study, participants discriminated the color of stimuli repeating or changing their location. Crucially, we varied the RSI from 500 ms to 3000 ms, trial-wise (Experiment 1) and block-wise (Experiment 2). We observed overall binding effects that were reduced with increasing RSI; these effects were slightly stronger when presented block-wise. IOR was overall absent (Experiment 1) or weak (Experiment 2) and did not emerge with increasing RSI. While event file decay took place, it did not unleash IOR. Rather, these results suggest that retrieval-based effects do not simply mask but overwrite IOR when manually responding. The observations of IOR with long intervals are discussed in the context of overall fast responding.

## Introduction

Every day we are confronted with numerous things that grab our attention – this may be our phone lighting up, an approaching car, or a large bird on a tree. Some of these things can be incorporated into our current actions, like picking up the phone to see if someone send a message. Thus, attention and action are often intertwined with each other when interacting with the environment. Yet, the relation between attention and action is still not fully understood (e.g., Lamy et al., in press; Frings et al., 2020; Henson et al., 2014).

One phenomenon that is thought to guide attention is labelled inhibition of return (IOR; Posner et al., 1985). When confronted with two stimuli in a sequence repeating or changing their location, IOR is marked by a slowed response to a previously attended location (for an overview, see Klein, 2000). This effect is thought to be resulting from an inhibition of said location (Posner & Cohen, 1984). While attentional disengagement of the previous location is not necessary for observing IOR (Berger et al., 2005) and the exact mechanisms are still debated (see, for example, Berlucchi, 2006; Dukewich & Klein, 2015), IOR has been observed numerous times, for example, when responding to the second of two stimuli (i.e., cue-target designs; e.g., Posner & Cohen, 1984), when responding to two stimuli in a sequence (i.e., target-target designs; e.g., Schöpper & Frings, 2024; Taylor & Donelly, 2002), when responding with eye movements (Kingstone & Pratt, 1999; Taylor & Klein, 2000; for a review, see Klein & Hilchey, 2011), or when responding to targets of different modalities (Schöpper & Frings, 2023; Spence et al., 2000).

The typical observations of IOR come from detection (e.g., Schöpper et al., 2020) and localization (e.g., Schöpper & Frings, 2022) procedures, where participants signal the detection or location of sequentially presented stimuli (see also Huffman et al., 2018; Schöpper & Frings, 2024). In contrast, for discrimination performance in which the non-spatial identity of sequentially presented stimuli repeating or changing their location is discriminated, the observation of IOR is less reliable. Albeit a co-occurrence is possible (Schöpper et al., 2020), several others have found no IOR if the to-be-discriminated target is repeated (Taylor & Ivanoff, 2005; Terry et al., 1994). It is typically argued that in such a case, repetition priming masks the occurrence of IOR (e.g., Klein, 2004; Taylor & Ivanoff, 2005; Terry et al., 1994). Yet, others have found that IOR can emerge in discrimination performance with longer stimulus onset asynchronies between cue and target compared to detection performance (Lupiáñez et al., 1997); in contrast, when cue identity has to be kept in working memory,^1^ IOR in discrimination performance onsets earlier than IOR resulting from localization performance (Gabay et al., 2012).

Hilchey et al. (2018) argued that the mixed results concerning the occurrence of IOR in discrimination performance are due to effects resulting from binding and retrieval in the realm of action control more strongly driving behavior in such tasks. When responding to a stimulus, action control theories like the theory of event coding (Hommel, 1998; Hommel et al., 2001) or the binding and retrieval in action control framework (BRAC; Frings et al., 2020; Frings, Beste, et al., 2024) assume that response and stimulus features are bound or integrated into a short episodic memory trace known as an event file (see Hommel, 2004). If now any component of the previous event file repeats, the previous information is retrieved, affecting performance: Partial repetition costs arise, marked by a cost of repeating the response but changing a response-irrelevant feature compared to a full repetition, as well as a cost of changing the response but repeating a response-irrelevant feature compared to a full change (Hommel, 1998). The resulting binding effects can be found in prime-probe sequences consisting of a prime display and a response to it, followed by a probe display and a response to it (Frings et al., 2007). Yet, the effects of binding and retrieval are thought to underlie many experimental paradigms that involve a sequential structure, like task switching (Koch et al., 2018), priming (Henson et al., 2014), conflict tasks (Davelaar & Stevens, 2009), and more (see Frings et al., 2020).

Based on that, Hilchey et al. (2018) hypothesized that binding effects should emerge when discriminating non-spatial target identity of a target, hiding the occurrence of IOR due to overlapping retrieval-based effects such as stimulus-response binding. In contrast, when simply orienting to the target, a discriminatory judgement is absent and thus should simply reveal IOR (see also Huffman et al., 2018). In their experiments, participants were instructed to make an eye movement to a target left or right of a fixation cross, followed by a manual discrimination of the target identity (+ or x) in a prime display. This was followed by the probe display with the same task. In two experiments, they observed IOR in saccadic movements; however, in manual responses, there were partial repetition costs, that is, binding effects. Hilchey et al. (2018) conclude that “inhibited reorienting is a ubiquitous side effect of prior oculomotor orienting, which can be completely overshadowed by integration effects” (p. 338). In line with that, binding effects are typically absent when detecting (Huffman et al., 2018; Schöpper et al., 2020) or localizing (Huffman et al., 2018; Schöpper & Frings, 2022; see, however, Schöpper et al., 2024, for binding effects in localization performance involving feature dimensions) stimuli repeating or changing their non-spatial identity and IOR is observed.^2^ Yet, when the task is to discriminate the nonspatial feature of a stimulus repeating or changing its location, strong binding effects between the discrimination response and location feature emerge (Hilchey et al., 2017; Huffman et al., 2018; Schöpper & Frings, 2024; Schöpper et al., 2020). Apart from this assumption of a sequential process, other interpretations are possible, for example, related to attentional, perceptual, and/or response-based processes. First, it has been argued that targets in discrimination tasks capture more attention (Klein, 2000), that the amount of attention towards non-spatial target identity is largely reduced in detection and localization performance (Huffman et al., 2018), and that the attentional disengagement of targets is prolonged (Lupiáñez et al., 2001; for a discussion, see Lupiáñez et al., 2013). Second, the efficiency of processing object representation might differ (Milliken et al., 2000; see also Lupiáñez, 2010; Lupiáñez et al., 2013). Third, discrimination responses have been argued to be affected by response repetition heuristics and/or repetition priming (Klein, 2004; Milliken et al., 2000; Taylor & Donelly, 2002; see also Schöpper & Frings, 2022, for a discussion).

Congruent with the assumption of a necessity for post-selective processing, Schöpper et al. (2022b) showed that binding effects can be observed in eye movements if the saccade is executed based on a discriminatory judgement. If the interval between prime response and probe display was short (ranging between 450–800 ms; Experiment 1), there was a binding effect, but no IOR in eye movement data. This is congruent with the suggestion by Hilchey et al. (2018) of retrieval-based effects masking IOR. Yet, if the interval was long (ranging between 1400–2000 ms; Experiment 2), there was no binding effect, but IOR. This is congruent with reduced or absent binding effects in manual movements if the interval between prime and probe gets too long, leading to event file decay that can no longer be retrieved (Frings, 2011; Hommel & Frings, 2020; Pastötter et al., 2021). Further, Schöpper et al. (2022b) argued that IOR might have been observed with such a long interval due to event file decay, causing IOR to no longer being masked by retrieval processes. If so, the question emerges, if the same applies for manually executed responses.

Congruent with that, IOR is usually absent when manually responding to sequentially presented central arrows pointing to the left or right (e.g., Taylor & Klein, 2000) – a task type resembling a shape discrimination judgement (Schöpper & Frings, 2022). However, with longer intervals between the presentation of arrow targets (i.e., exceeding 2000 ms), cueing effects indicated by a location repetition cost^3^ can be observed (Avery et al., 2015; Ding et al., 2016; Weger et al., 2008; for a discussion, see Schöpper & Frings, 2022, pp. 652–653) – likely caused by the attention-shifting properties of arrow shapes (e.g., Pratt & Hommel, 2003; Pratt et al., 2010). This again suggests that for long intervals, retrieval-based effects have declined and are no longer strong enough to mask IOR or inhibitory effects alike in manual responses as well.

However, it is also possible that IOR resulting from manual or eye movements is differently affected by reduced effects of retrieval, for example, due to the involvement of specific oculomotor mechanisms for the latter (e.g., Kingstone & Pratt, 1999; Taylor & Klein, 2000; for a review, see Klein & Hilchey, 2011). That is, only IOR in eye movements (e.g., Hilchey et al., 2018; Schöpper et al., 2022b) caused by prior oculomotor orienting might resurface after retrieval-based effects have decayed – whereas for manual movements without a strong involvement of the oculomotor system IOR does not emerge after event file decay.

## Current study

We hypothesized that it is possible to observe IOR in manual discrimination responses if the interval between prime and probe is very long and as a consequence event files decay and no longer mask IOR. This would be in line with retrieval-based effects masking IOR during short intervals (Hilchey et al., 2018) but no longer during long intervals (Schöpper et al., 2022b) found with eye movements. We adopted the procedure of the discrimination tasks used in Schöpper et al. (2020): Participants were instructed to discriminate the color of targets repeating or changing their location. In turn, color becomes response-relevant, whereas location is response-irrelevant. In two experiments, Schöpper et al. (2020) observed overall IOR, that is, a location repetition cost, accompanied by an interaction of color response and location: Color response repetitions were impaired by changing the response-irrelevant location and color response changes were impaired by repeating the response-irrelevant location. In other words, they observed strong binding effects as well as IOR. In the current study, we used such a discrimination task but varied the response-stimulus interval (RSI) to range between 500 ms and 3000 ms. We expect to observe an overall binding pattern between color response^4^ and location (cf. Hilchey et al., 2017) that should decay with increasing RSI (Frings, 2011; Hommel & Frings, 2020); in other words, the effect should be pronounced at the shortest RSIs. In contrast, we expect to observe overall IOR that should increase in size with increasing RSI due to no longer being masked by retrieval. Whereas event files decay very quick, IOR is stable up to 3000 ms (Samuel & Kat, 2003) and should be pronounced at the longest RSIs. The decay function was tested trial-wise (Experiment 1) and block-wise (Experiment 2).

In the design by Schöpper et al. (2020) targets are always presented at an upper or lower position in the right screen half. While it is possible that responding might be influenced by target position (Snyder & Schmidt, 2014) which might even have an impact on the strength of IOR (Soballa et al., 2022), this setup has been found to yield overall IOR (Schöpper et al., 2020) and gives the advantage of reducing the impact of Simon-like interference (e.g., Simon, 1969; Simon & Small, 1969; see also Hommel, 2011), that is, we circumvent that left/right response keys spatially correspond to horizontally presented targets (albeit an influence of orthogonal stimulus-response compatibility is still possible, Nishimura & Yokosawa, 2006; cf. Lippa & Adam, 2001).

Of note, if binding and retrieval occurs in a color discrimination task with location as response-irrelevant feature, a location repetition cost would manifest at response changes – interference caused by the location retrieving the now incorrect response. While it has been argued that IOR can be differently affected by repeating or changing stimulus information in discrimination tasks (e.g., Chao et al., 2022; Taylor & Donelly, 2002; Taylor & Ivanoff, 2005; Terry et al., 1994) and that response-repetition heuristics (e.g., Pashler & Baylis, 1991) might overlap IOR when continuously responding to targets (Klein, 2004), these location repetition costs could also simply reflect partial repetition costs. In short, for response changes it is not possible to disentangle, if location repetition costs in these are caused by retrieval, IOR, or both (for a similar discussion, see Schöpper & Frings, 2022, p. 650; see also Chao & Hsiao, 2021; Chao et al., 2022). Importantly, we were interested in an overall location repetition cost that occurs irrespective of repeating or changing the response (e.g., Hilchey et al., 2018; Schöpper et al., 2020; Taylor & Donelly, 2002). However, note that effects of response/color repetition might be interpreted to further modulate this pattern (cf. Taylor & Donelly, 2002).

## Experiment 1

### Methods

#### Participants

In Schöpper et al. (2020), the binding effects between response and location were very strong, coming with effects sizes above *d* > 2.5 (*d* = 2.97 in Experiment 1, and *d* = 2.77 in Experiment 2; see also Schöpper & Frings, 2024). IOR is a stable effect as well, coming with medium to high effect sizes in detection and localization performance (e.g., *d* = 1.14 in Schöpper & Frings, 2022; see also Schöpper & Frings, 2024). In discrimination performance, IOR can sometimes be completely absent in the data (e.g., Hilchey et al., 2018; Schöpper et al., 2022b) – as introduced it is potentially overlapped by retrieval processes – or a co-occurrence can be observed in which IOR can reach medium to high effect sizes (e.g., *d* = 0.70 in Experiment 1, and *d* = 0.63 in Experiment 2 in Schöpper et al., 2020). We decided to collect data for *N* = 30 participants. In turn, 30 students of the University of Trier (26 female, 4 male; *M_Age_* = 24.20; *SD_Age_* = 4.16; age range 19–33 years) participated for either course credit or a monetary reward (15 Euro). Under α = .05 (one-tailed) this sample size yields a power of 1–β = .98 for detecting an IOR effect of at least *d* = 0.70 and a power 1–β = 1.00 for detecting a binding effect of response and location of at least *d* = 2.70 (G*Power, Version 3.1.9.2; Faul et al., 2007). The experiment was conducted in accordance with ethical guidelines of the University of Trier and all participants gave written informed consent. All participants reported normal or corrected-to-normal vision.

#### Apparatus and Materials

The experiment geared to the discrimination tasks used in Schöpper et al. (2020) and Schöpper and Frings (2024); however, stimuli were larger in size compared to both previous studies. The experiment was programmed in PsychoPy (Peirce et al., 2019) and was presented on a monitor with a display resolution of 1680 × 1050 px (length × height: 44.45° × 28.63° of visual angle derived from a distance of 58 cm; however, we did not use a chin rest, so perceived sizes may have varied). A white (R/G/B: 255/255/255) fixation cross (0.89° × 0.89°) was presented in the left screen half on a black (R/G/B: 0/0/0) background. Targets were circles 1.38° in diameter and were red (R/G/B: 224/32/64) or blue (R/G/B: 64/64/192). These targets appeared in the right screen half, 11.13° away from the fixation cross on the x-axis and 4.15° above or below it.

#### Design

The experiment used a 6 (RSI: 500 × 1000 × 1500 × 2000 × 2500 × 3000 ms) × 2 (response relation: repetition vs. change) × 2 (location relation: repetition vs. change) design. All variables were varied within subjects. An IOR effect can be derived from the main effect of location relation, depicting a location repetition cost. A binding effect between response and location can be derived from the interaction of response relation and location relation, depicting partial repetition costs. Their modulations are derived from the interactions with RSI.

#### Procedure

**Figure 1:**
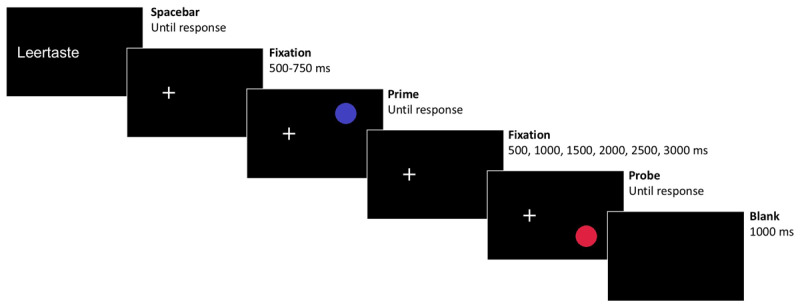
An exemplary trial sequence (not drawn-to-scale), depicting a trial with both the color – and by that the response – and location changing.

Targets were presented in prime-probe sequences: Participants first saw a prime target and gave a response to it, which was followed by a probe target and a response to it. A trial started with the presentation of the german word “Leertaste” (Spacebar) on the left side; participants started each prime-probe sequence by pressing the spacebar. This was followed by the fixation cross appearing on the left, remaining for the whole sequence until probe response execution; participants were instructed to fixate throughout. After a variable fixation interval of 500–750 ms, the prime stimulus – a red or blue circle – appeared on the right side of the screen at either the top or bottom position. Participants were instructed to press the f-key for blue targets (with the left index finger) and the j-key for red targets (with the right index finger) irrespective of their location. Targets remained visible until response. After the prime display, the fixation cross was shown in isolation for a duration of 500 ms, 1000 ms, 1500 ms, 2000 ms, 2500 ms, or 3000 ms, depending on the RSI of the respective trial. This was followed by the probe target, to which a discrimination response had to be made as outlined for the prime display. After the probe display, the display turned blank for 1000 ms, ending a prime-probe sequence (see Figure 1). If participants pressed the wrong key in the prime or probe display or gave a response during the fixation screen prior to the probe display (i.e., during the RSI), an error message appeared directly after the incorrect response for 1000 ms (“Falsch!”, wrong, and “Zu früh!”, too early, respectively).

From prime to probe the color and by that the response could repeat (response repetition, RR) or change (response change, RC), and the location of the target could repeat (location repetition, LR) or change (location change, LC). These factors were orthogonally varied and occurred equally often with each of the six RSIs. All combinations of response, location, and RSI were pseudo-randomly balanced, and conditions were drawn randomly. The experiment started with 16 practice trials drawn randomly from the set of all different combinations, and participants received feedback after every response. This was followed by the experiment with 28 trials for every condition, that is, 672 trials in total. Here, participants received feedback only for incorrect responses. After every 28^th^ experimental trial participants could take a self-paced break.

### Results

#### Reaction times

Reaction times above 200 ms and below 1.5 interquartile range above the third quartile of a participant’s distribution (Tukey, 1977) were included for analysis. Additionally, we only included trials in which both prime and probe response were correct as well as no response was given during the fixation interval. This led to 13.77% of trials being discarded.

A 6 (RSI: 500 × 1000 × 1500 × 2000 × 2500 × 3000 ms) × 2 (response relation: repetition vs. change) × 2 (location relation: repetition vs. change) repeated-measures MANOVA^5^ on probe reaction times (Table 1) revealed a main effect of response relation, *F*(1, 29) = 39.34, *p* < .001, \[
\eta_p^2
\] = .58, with participants being faster if the response repeated (492 ms) compared to changed (520 ms). Overall IOR was absent as suggested by the not significant main effect of location relation, *F*(1, 29) = 0.78, *p* = .383, \[
\eta_p^2
\] = .03, and the main effect of RSI was not significant as well, *F*(5, 25) = 1.87, *p* = .135, \[
\eta_p^2
\] = .27. Interestingly, RSI did also not modulate location relation, *F*(5, 25) = 0.59, *p* = .710, \[
\eta_p^2
\] = .11. Yet, RSI modulated response relation, *F*(5, 25) = 14.74, *p* < .001, \[
\eta_p^2
\] = .75: The response repetition benefit became smaller with increasing RSI, starting at 500 ms (RR: 482 ms; RC: 533 ms), followed by 1000 ms (RR: 479 ms; RC: 516 ms), 1500 ms (RR: 488 ms; RC: 519 ms), 2000 ms (RR: 499 ms; RC: 520 ms), 2500 ms (RR: 499 ms; RC: 515 ms), and 3000 ms (RR: 503 ms; RC: 513 ms). There was an interaction of response relation and location relation, *F*(1, 29) = 75.98, *p* < .001, \[
\eta_p^2
\] = .72, suggesting partial repetition costs: Participants were slower in response repetitions when the location changed (499 ms) compared to repeated (484 ms). In contrast, participants were slower in response changes when the location repeated (529 ms) compared to changed (510 ms). Crucially, this interaction was further modulated by RSI, *F*(5, 25) = 2.78, *p* = .040, \[
\eta_p^2
\] = .36.

**Table 1:** Mean reaction times in milliseconds and error rates in percent (in brackets) of probe responses for each condition and RSI, separate for Experiment 1 and Experiment 2. RR = Response repetition, RC = Response change, LR = Location repetition, LC = Location change.


EXPERIMENT	RSI	RRLR	RRLC	RCLR	RCLC

Experiment 1	500	472 (0.51)	492 (2.71)	549 (5.74)	518 (2.03)

	1000	468 (1.73)	490 (2.96)	528 (6.93)	504 (1.96)

	1500	479 (2.60)	497 (4.26)	529 (4.74)	508 (2.63)

	2000	497 (2.36)	502 (6.00)	528 (4.37)	513 (3.19)

	2500	490 (3.82)	507 (5.96)	522 (4.48)	509 (4.25)

	3000	498 (3.39)	507 (5.52)	516 (5.72)	511 (3.62)

Experiment 2	500	400 (0.51)	439 (4.99)	474 (8.55)	428 (1.12)

	1000	422 (1.18)	444 (5.14)	480 (6.69)	440 (2.01)

	1500	450 (1.53)	471 (4.45)	499 (4.50)	472 (2.35)

	2000	479 (2.89)	486 (5.41)	505 (4.12)	482 (2.78)

	2500	474 (2.33)	496 (4.82)	511 (3.83)	488 (3.99)

	3000	490 (3.72)	507 (4.80)	509 (5.46)	498 (3.32)


**Figure 2:**
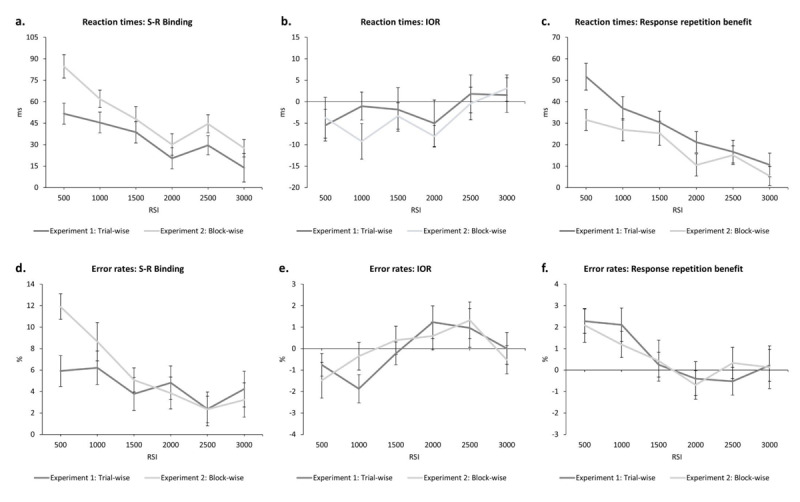
**a)** The calculated S-R binding effects depending on RSI in reaction times. **b)** The calculated IOR effects depending on RSI in reaction times. **c)** The calculated response repetition benefit depending on RSI in reaction times. **d)** The calculated S-R binding effects depending on RSI in error rates. **e)** The calculated IOR effects depending on RSI in error rates. **f)** The calculated response repetition benefit depending on RSI in error rates. The dark grey lines depict the results of Experiment 1 (trial-wise presentation of RSI) and the light grey lines depict the results of Experiment 2 (block-wise presentation of RSI). All error bars represent standard error of each mean.

Due to the significant threeway interaction we calculated the binding effects for each RSI – the interaction of response relation and location relation – via (RRLC-RRLR)-(RCLC-RCLR), which sums up the partial repetition costs of response repetitions and response changes, respectively (see Frings, 2011; Schöpper & Frings, 2022, 2024). These differential values correspond to the interaction of RSI, response relation, and location relation. The calculated effects showed a linear trend, *F*(1, 29) = 10.45, *p* = .003, \[
\eta_p^2
\] = .27: The binding effect was strongest at the shortest RSI and declined with increasing RSI (500: 52 ms; 1000: 45 ms; 1500: 39 ms; 2000: 20 ms; 2500: 30 ms; 3000: 14 ms; see Figure 2a).

For sake of completeness, we calculated IOR for each RSI – the main effect of location relation – via ((RRLC+RCLC)/2)-((RRLR+RCLR)/2), which leads to a location repetition cost in case of IOR (Schöpper & Frings, 2024; cf. Schöpper & Frings, 2022). Additionally, we did the same for response repetitions via ((RCLR+RCLC)/2)-((RRLR+RRLC)/2), with a positive value resembling a response repetition benefit (e.g., Schöpper & Frings, 2024). The differential values for IOR (500: –5 ms; 1000: –1 ms; 1500: –2 ms; 2000: –5 ms; 2500: 2 ms; 3000: 2 ms) correspond to the interaction of RSI and location relation and are depicted in Figure 2b. The values for the response repetition benefit (500: 52 ms; 1000: 37 ms; 1500: 30 ms; 2000: 21 ms; 2500: 17 ms; 3000: 11 ms) correspond to the interaction of RSI and response relation, are depicted in Figure 2c, and depicted a linear trend, *F*(1, 29) = 63.13, *p* < .001, \[
\eta_p^2
\] = .69. This suggests that the response repetition benefit was reduced with increasing RSI. All effects tested individually for each RSI can be found in Appendix A1.

#### Error rates

Error rate is the percentage of incorrect probe responses after correct prime responses. We excluded trials in which the prime response was correct or if a response was given during the fixation interval. This led to 6.46% of trials being discarded.

A 6 (RSI: 500 × 1000 × 1500 × 2000 × 2500 × 3000 ms) × 2 (response relation: repetition vs. change) × 2 (location relation: repetition vs. change) repeated-measures MANOVA^6^ on probe error rates (Table 1) revealed no main effects of response relation, *F*(1, 29) = 3.10, *p* = .089, \[
\eta_p^2
\] = .10, location relation, *F*(1, 29) = 0.20, *p* = .657, \[
\eta_p^2
\] = .01, or the main effect of RSI, *F*(5, 25) = 2.45, *p* = .062, \[
\eta_p^2
\] = .33. RSI did not modulate location relation, *F*(5, 25) = 2.34, *p* = .071, \[
\eta_p^2
\] = .32, but modulated response relation, *F*(5, 25) = 3.64, *p* = .013, \[
\eta_p^2
\] = .42: The response repetition benefit mainly occurred at the earliest RSIs, starting with 500 ms (RR: 1.61%; RC: 3.88%), followed by 1000 ms (RR: 2.34%; RC: 4.45%), 1500 ms (RR: 3.43%; RC: 3.69%), 2000 ms (RR: 4.18%; RC: 3.78%), 2500 ms (RR: 4.89%; RC: 4.36%), and 3000 ms (RR: 4.46%; RC: 4.67%). There was an interaction of response relation and location relation, *F*(1, 29) = 23.20, *p* < .001, \[
\eta_p^2
\] = .44, depicting partial repetition costs (RRLR: 2.40%: ; RRLC: 4.57%; RCLR: 5.33%; RCLC: 2.95%). Interestingly, this interaction was not modulated by RSI, *F*(5, 25) = 1.05, *p* = .410, \[
\eta_p^2
\] = .17.

Calculated binding effects (500: 5.91%; 1000: 6.21%; 1500: 3.77%; 2000: 4.82%; 2500: 2.37%; 3000: 4.23%) are depicted in Figure 2d. For the calculated IOR effects the linear trend, *F*(1, 29) = 7.05, *p* = .013, \[
\eta_p^2
\] = .20, and the cubic trend, *F*(1, 29) = 5.12, *p* = .031, \[
\eta_p^2
\] = .15, became significant (500: –0.76%; 1000: –1.87%; 1500: –0.23%; 2000: 1.23%; 2500: 0.96%; 3000: 0.01%; see Figure 2e). For response repetition benefits (500: 2.27%; 1000: 2.10%; 1500: 0.26%; 2000: –0.40%; 2500: –0.53%; 3000: 0.21%; see Figure 2f), the linear trend, *F*(1, 29) = 11.15, *p* = .002, \[
\eta_p^2
\] = .28, and the quadratic trend, *F*(1, 29) = 5.72, *p* = .024, \[
\eta_p^2
\] = .17, became significant. All effects tested individually for each RSI can be found in Appendix A2.

#### Discussion

Participants responded to the color of targets repeating or changing their location. Between the targets were different RSIs. As expected, there was a binding pattern between response and location, suggesting partial repetition costs. Congruent with previous research (e.g., Frings, 2011; Hommel & Frings, 2020) event files decayed with more time between prime response and probe onset in reaction times. Interestingly, binding effects were still observable at the longest RSIs of 3000 ms. Further, in error rates the binding effects remained stable across RSIs. In contrast, overall IOR was absent, being congruent with this effect being masked by retrieval (Hilchey et al., 2018; Schöpper et al., 2022b). However, IOR did also not emerge with increasing RSIs: Although event files comprising color response and location information decayed, this decay did not unleash IOR. In contrast, there was even a mild trend of a location repetition cost occurring only in the shortest RSIs in error rates. Lastly, an overall response repetition benefit was most prominently observable at the shortest RSIs and decayed with increasing RSI as well. To summarize, we found binding effects and their decrease in size with increasing RSI. Yet, IOR was absent and did not emerge with decreasing retrieval-based effects.

## Experiment 2

Several of previous observations of present or absent IOR depending on RSI stem from individually conducted studies with not much variance in RSIs in each (e.g., only short and long RSIs in different experiments in Schöpper et al., 2022b; only short intervals in Schöpper & Frings, 2022, and only long intervals in Avery et al., 2015; see, however, e.g., Gabay et al., 2012). We thus thought it prudent to replicate our results in a second experiment with blocks of constant RSIs.

### Methods

#### Participants

In Experiment 2, 30 students (24 female, 6 male; *M_Age_* = 24.53; *SD_Age_* = 2.35; age range 20–29 years) of the University of Trier participated for either course credit or a monetary reward (15 Euro) and gave written informed consent. None had participated in Experiment 1. One participant reported an uncorrected color blindness; however, the data was inconspicuous when compared with the sample and was thus included in the analysis. All other participants reported normal or corrected-to-normal vision.

#### Apparatus, Materials, Design, and Procedure

Experiment 2 was identical to Experiment 1 except for the following: RSIs were not picked trial-wise, but participants worked through six blocks with constant RSIs, each. The order of the six RSIs was picked randomly for each participant. In practice trials at the start of the experiment, 16 trials were picked at random from all possible combinations including variable RSIs (i.e., only in the practice trials the RSI could change trial-wise).

### Results

#### Reaction times

We used the same criteria as in Experiment 1. This led to 12.85% of trials being discarded. A 6 (RSI: 500 × 1000 × 1500 × 2000 × 2500 × 3000 ms) × 2 (response relation: repetition vs. change) × 2 (location relation: repetition vs. change) repeated-measures MANOVA^7^ on probe reaction times (Table 1) revealed a main effect of response relation *F*(1, 29) = 28.98, *p* < .001, \[
\eta_p^2
\] = .50 (RR: 463 ms; RC: 482 ms). This time, the main effect of location relation was significant, *F*(1, 29) = 4.45, *p* = .044, \[
\eta_p^2
\] = .13: Participants were slower when a target repeated its location (474 ms) compared to changing it (471 ms), suggesting IOR. The main effect of RSI was significant as well, *F*(5, 25) = 32.25, *p* < .001, \[
\eta_p^2
\] = .87: Participants became slower with increasing RSI (500: 435 ms; 1000: 446 ms; 1500: 473 ms; 2000: 488 ms; 2500: 492 ms; 3000: 501 ms). Again, RSI did not modulate location relation, *F*(5, 25) = 2.10, *p* = .100, \[
\eta_p^2
\] = .30; however, RSI modulated response relation, *F*(5, 25) = 10.25, *p* < .001, \[
\eta_p^2
\] = .67, again showing the decrease of the response repetition benefit from short to long RSI, starting at 500 ms (RR: 420 ms; RC: 451 ms), followed by 1000 ms (RR: 433 ms; RC: 460 ms), 1500 ms (RR: 460 ms; RC: 486 ms), 2000 ms (RR: 483 ms; RC: 493 ms), 2500 ms (RR: 485 ms; RC: 500 ms), and 3000 ms (RR: 498 ms; RC: 504 ms). There was an interaction of response relation and location relation, *F*(1, 29) = 149.05, *p* < .001, \[
\eta_p^2
\] = .84, suggesting partial repetition costs (RRLR: 453 ms; RRLC: 474 ms; RCLR: 496 ms; RCLC: 468 ms). Importantly, this interaction was further modulated by RSI, *F*(5, 25) = 13.62, *p* < .001, \[
\eta_p^2
\] = .73.

Calculated binding effects were marked by a linear trend, *F*(1, 29) = 57.62, *p* < .001, \[
\eta_p^2
\] = .67, and quadratic trend, *F*(1, 29) = 5.34, *p* = .028, \[
\eta_p^2
\] = .16: The binding effect was strongest at the shortest RSI and declined with increasing RSI (500: 85 ms; 1000: 62 ms; 1500: 48 ms; 2000: 30 ms; 2500: 45 ms; 3000: 27 ms; see Figure 2a).

Calculated IOR-effects (500: –4 ms; 1000: –9 ms; 1500: –3 ms; 2000: –8 ms; 2500: 0 ms; 3000: 3 ms) are depicted in Figure 2b. Response repetition benefits (500: 31 ms; 1000: 27 ms; 1500: 25 ms; 2000: 10 ms; 2500: 15 ms; 3000: 5 ms; see Figure 2c) were marked by a linear trend, *F*(1, 29) = 29.53, *p* < .001, \[
\eta_p^2
\] = .51. All effects tested individually for each RSI can be found in Appendix A1.

#### Error rates

We excluded trials in which the prime response was correct or if a response was given during the fixation interval. This led to 5.74% of trials being discarded.

A 6 (RSI: 500 × 1000 × 1500 × 2000 × 2500 × 3000 ms) × 2 (response relation: repetition vs. change) × 2 (location relation: repetition vs. change) repeated-measures MANOVA^8^ on probe error rates (Table 1) revealed no main effects of response relation, *F*(1, 29) = 1.87, *p* = .182, \[
\eta_p^2
\] = .06, location relation, *F*(1, 29) < .01, *p* = .972, \[
\eta_p^2
\] = .00, or the main effect of RSI, *F*(5, 25) = 0.83, *p* = .541, \[
\eta_p^2
\] = .14. RSI did not modulate response relation, *F*(5, 25) = 1.57, *p* = .206, \[
\eta_p^2
\] = .24, or location relation, *F*(5, 25) = 1.85, *p* = .140, \[
\eta_p^2
\] = .27. There was an interaction of response relation and location relation, *F*(1, 29) = 45.68, *p* < .001, \[
\eta_p^2
\] = .61, depicting partial repetition costs (RRLR: 2.03%; RRLC: 4.93%; RCLR: 5.52%; RCLC: 2.59%). Crucially, this interaction was modulated by RSI, *F*(5, 25) = 8.94, *p* < .001, \[
\eta_p^2
\] = .64.

For the calculated binding effects the linear trend, *F*(1, 29) = 31.76, *p* < .001, \[
\eta_p^2
\] = .52, and the quadratic trend, *F*(1, 29) = 8.09, *p* = .008, \[
\eta_p^2
\] = .22, became significant. This depicted a decrease of a binding pattern with increasing RSI (500: 11.91%; 1000: 8.64%; 1500: 5.07%; 2000: 3.85%; 2500: 2.33%; 3000: 3.22%; see Figure 2d).

For IOR, the quadratic trend became significant, *F*(1, 29) = 7.28, *p* = .012, \[
\eta_p^2
\] = .20 (500: –1.48%; 1000: –0.36%; 1500: 0.39%; 2000: 0.59%; 2500: 1.32%; 3000: –0.53%; see Figure 2e). Response repetition benefits (500: 2.08%; 1000: 1.19%; 1500: 0.43%; 2000: –0.70%; 2500: 0.33%; 3000: 0.13%) are depicted in Figure 2f. All effects tested individually for each RSI can be found in Appendix A2.

#### Discussion

In the second experiment, participants performed the same color discrimination task, except for RSI being modulated block-wise. Again, there was a binding pattern between response and location, which decreased in size with increasing RSI both in reaction times and error rates. This time, overall IOR emerged but was unmodulated by RSI. For sake of completeness, we conducted a between-experiment analysis to see if decay rates were different between experiments.

### Between experiment analysis

#### Reaction times

A repeated measures MANOVA with RSI (500 × 1000 × 1500 × 2000 × 2500 × 3000 ms) and experiment (1: trialwise vs. 2: blockwise) on the calculated binding effects revealed a significant main effect of RSI, *F*(5, 54) = 11.51, *p* < .001, \[
\eta_p^2
\] = .52, coming with a linear trend, *F*(1, 58) = 44.27, *p* < .001, \[
\eta_p^2
\] = .43, depicting a decrease of a binding pattern with increasing RSI (500: 68 ms; 1000: 54 ms; 1500: 43 ms; 2000: 25 ms; 2500: 37 ms; 3000: 21 ms). The main effect of experiment was significant as well, *F*(1, 58) = 8.43, *p* = .005, \[
\eta_p^2
\] = .13, showing larger overall effects in Experiment 2 (49 ms) compared to Experiment 1 (33 ms). However, experiment and RSI did not interact, *F*(5, 54) = 0.73, *p* = .605, \[
\eta_p^2
\] = .06. The same MANOVA on the calculated IOR effects showed no significant effects (all *F* ≤ 1.79; all *p* ≥ .131). A more detailed 2 (Experiment: Trialwise vs. Blockwise) × 6 (RSI: 500 × 1000 × 1500 × 2000 × 2500 × 3000 ms) × 2 (response relation: repetition vs. change) × 2 (location relation: repetition vs. change) repeated-measures MANOVA on probe reaction times is reported in Appendix A3.

#### Error rates

A repeated measures MANOVA with RSI (500 × 1000 × 1500 × 2000 × 2500 × 3000 ms) and experiment (1: trialwise vs. 2: blockwise) on the calculated binding effects revealed a significant main effect of RSI, *F*(5, 54) = 6.39, *p* < .001, \[
\eta_p^2
\] = .37, coming with a linear trend, *F*(1, 58) = 25.41, *p* < .001, \[
\eta_p^2
\] = .31, and quadtratic trend, *F*(1, 58) = 5.80, *p* = .019, \[
\eta_p^2
\] = .09, depicting a decrease of a binding pattern with increasing RSI (500: 8.91%; 1000: 7.43%; 1500: 4.42%; 2000: 4.34%; 2500: 2.35%; 3000: 3.72%). The main effect of experiment was not significant, *F*(1, 58) = 1.01, *p* = .319, \[
\eta_p^2
\] = .02. Experiment and RSI did not interact, *F*(5, 54) = 2.03, *p* = .090, \[
\eta_p^2
\] = .16. Yet, the linear trend of this interaction was significant, *F*(1, 58) = 7.47, *p* = .008, \[
\eta_p^2
\] = .11, suggesting that the decay rate in the block-wise experiment was slightly steeper. The same MANOVA on the calculated IOR effects showed a main effect of RSI, *F*(5, 54) = 3.60, *p* = .007, \[
\eta_p^2
\] = .25, coming with a linear trend, *F*(1, 58) = 7.41, *p* = .009, \[
\eta_p^2
\] = .11, quadratic trend, *F*(1, 58) = 56.95, *p* = .011, \[
\eta_p^2
\] = .11, and a cubic trend, *F*(1, 58) = 4.82, *p* = .032, \[
\eta_p^2
\] = .08, roughly depicting a location repetition cost at the earliest RSIs turning into descriptive location repetition benefits at later RSIs (500: –1.12%; 1000: –1.11%; 1500: 0.08%; 2000: 0.91%; 2500: 1.14%; 3000: –0.26%). All other effects were not significant (all *F* ≤ 1.79; all *p* ≥ .131). A more detailed 2 (Experiment: Trialwise vs. Blockwise) × 6 (RSI: 500 × 1000 × 1500 × 2000 × 2500 × 3000 ms) × 2 (response relation: repetition vs. change) × 2 (location relation: repetition vs. change) repeated-measures MANOVA on probe reaction times is reported in Appendix A4.

#### Discussion

The between-experiment analysis revealed that decay rates for event files were comparable across experiments, with a slight tendency for stronger binding effects (reaction times) and steeper decay functions (error rates) when RSIs were varied block-wise. This suggests that event file decay is not a general, passive process but rather depends on the context set by temporal characteristics of the experimental sequence (cf. Horoufchin et al., 2011; see also Mondor & Leboe, 2008). Future studies could systematically look at the impact of predictable or unpredictable temporal information on current decay rates (see Horoufchin et al., 2011). Overall IOR was present but did not emerge with increasing RSIs; rather, the analysis of error rates revealed a location repetition cost in the earliest RSIs – when retrieval-based effects were strongest(!) – which disappeared and even descriptively turned into a location repetition benefit with increasing RSIs.

### Explorative analysis: Impact of response speed

Whereas Schöpper et al. (2020) found strong IOR effects in two experiments, we only found a small effect in Experiment 2 and none in Experiment 1. At first, this is at odds, as we used nearly the exact same design, except for the following most striking differences: The original study had a constant RSI of 500 ms (current study: variable with a range between 500–3000 ms), presented (smaller) targets only for 100 ms with responding being possible up to 1100 ms (current study: presented until response), and used catch trials (current study: no catch trials). However, one can notice that participants in Schöpper et al. (2020) were drastically faster (Experiment 1: 412 ms; Experiment 2: 395 ms) compared to the current study (Experiment 1: 506 ms; Experiment 2: 473 ms); additionally, in the current study we only found IOR in Experiment 2, in which participants overall responded 33 ms faster compared to Experiment 1 with absent IOR. IOR has been found to emerge earlier in time than retrieval-based effects (cf. Chao et al., 2020, 2022; Schöpper & Frings, 2022, 2024); yet, with more time for responding, retrieval-based effects take over (see also Schöpper & Frings, 2024). Thus, it is possible that in Schöpper et al. (2020) and Experiment 2 of the current study IOR was observed because participants were simply overall faster, allowing early-onsetting IOR to manifest. In contrast, if overall responding is slow, IOR is completely overwritten by established retrieval.

**Figure 3:**
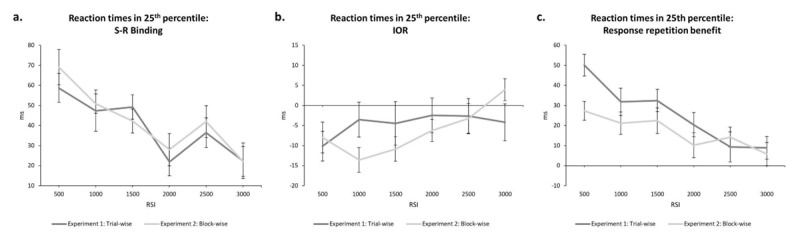
**a)** The calculated S-R binding effects depending on RSI in reaction times in the 25^th^ percentile rank. **b)** The calculated IOR effects depending on RSI in reaction times in the 25^th^ percentile rank. **c)** The calculated response repetition benefit depending on RSI in reaction times in the 25^th^ percentile rank. The dark grey lines depict the results of Experiment 1 (trial-wise presentation of RSI) and the light grey lines depict the results of Experiment 2 (block-wise presentation of RSI). All error bars represent standard error of each mean.

To test for this, we took the 25^th^ percentile rank of reaction times of every condition for every participant separate for each experiment. Based on previous studies (cf. Schöpper & Frings, 2022, 2023, 2024), we expected to find strong IOR and weaker effects of retrieval in these early responses in both experiments. We then conducted the 6 (RSI: 500 × 1000 × 1500 × 2000 × 2500 × 3000 ms) × 2 (response relation: repetition vs. change) × 2 (location relation: repetition vs. change) repeated-measures MANOVA on the 25^th^ percentile of probe reaction times. In Experiment 1, this revealed a main effect of location relation, *F*(1, 29) = 4.25, *p* = .048, \[
\eta_p^2
\] = .13, suggesting the occurrence of IOR (LR: 442 ms; LC: 438 ms). Yet, this effect was unmodulated by RSI, *F*(1, 29) = 0.79, *p* = .568, \[
\eta_p^2
\] = .14 (see Figure 3b). In contrast, the binding effect of response and location, *F*(1, 29) = 90.22, *p* < .001, \[
\eta_p^2
\] = .76, (RRLR: 420 ms; RRLC: 435 ms; RCLR: 465 ms; RCLC: 441 ms) decreased with increasing RSI, *F*(1, 29) = 3.20, *p* = .023, \[
\eta_p^2
\] = .39 (see Figure 3a). The same held true for the response repetition benefit, *F*(1, 29) = 26.38, *p* < .001, \[
\eta_p^2
\] = .48 (RR: 427 ms; RC: 453 ms), which was reduced with increasing RSI, *F*(1, 29) = 15.19, *p* < .001, \[
\eta_p^2
\] = .75 (see Figure 3c). In Experiment 2, the main effect of location relation was significant as well, *F*(1, 29) = 12.34, *p* = .001, \[
\eta_p^2
\] = .30, again depicting IOR (LR: 418 ms; LC: 412 ms). This time, IOR was modulated by RSI, *F*(1, 29) = 4.14, *p* = .007, \[
\eta_p^2
\] = .45, suggesting that the effect decreased with increasing RSI (see Figure 3b). The binding effect of response and location was significant, *F*(1, 29) = 100.05, *p* < .001, \[
\eta_p^2
\] = .78 (RRLR: 399 ms; RRLC: 414 ms; RCLR: 437 ms; RCLC: 410 ms), and decreased with increasing RSI, *F*(1, 29) = 5.29, *p* = .002, \[
\eta_p^2
\] = .51 (see Figure 3a). Lastly, the response repetition benefit was significant, *F*(1, 29) = 26.38, *p* < .001, \[
\eta_p^2
\] = .48 (RR: 407 ms; RC: 424 ms), and was significantly reduced with increasing RSI, *F*(1, 29) = 5.91, *p* < .001, \[
\eta_p^2
\] = .54 (see Figure 3c). This explorative analysis reveals that if responding is fast, IOR is evident; if responses get slower, binding and retrieval take over (cf. Schöpper & Frings, 2024) increasingly overwriting IOR. Yet, even when IOR is strong (i.e., in early responses), it does not increase in size with increasing RSI – in Experiment 2 even the opposite – although in parallel event file decay takes place.

## General Discussion

In the current study, participants discriminated the color of targets repeating or changing their location. As expected, we observed a strong stimulus-response binding pattern between the color response and location feature, marked by partial repetition costs in both reaction times and error rates. These binding effects decreased in size with increasing RSI, congruent with event file decay (Frings, 2011; Hommel & Frings, 2020; Pastötter et al., 2021; Schöpper et al., 2022b) reducing the impact of retrieval on responding. In contrast, IOR was absent in Experiment 1, and only weak in Experiment 2. Further, it did not emerge with increasing RSI.

It has previously been assumed that retrieval-based effects *mask* IOR (Hilchey et al., 2018; Klein, 2004; Schöpper & Frings, 2022; Schöpper et al., 2022b; Taylor & Ivanoff, 2005; Terry et al., 1994). If this would be the case, we would have observed a strengthening of IOR with increasing event file decay, due to no longer being masked by retrieval. Yet, this pattern was absent as IOR did not increase in size. If anything, there was a tendency for IOR only being present at the earliest RSIs – when retrieval was very strong. Rather, the results suggest that retrieval-based effects *overwrite* IOR for manual responses. In the same vein, an overall response repetition benefit also declined with increasing RSI, suggesting that potentially occurring response repetition heuristics might not only hide IOR (e.g., Klein, 2004), but also overwrite it.

The question then emerges, why there are some cases of observed IOR with long intervals between stimuli in discrimination tasks. Schöpper et al. (2022b) found IOR and no binding pattern with a long interval between saccadic discrimination responses. In contrast, the current study only used manual responses. Thus, it could be that different types of IOR emerged depending on the involvement of the oculomotor system (Klein & Redden, 2018; Redden et al., 2021). In fact, saccadic IOR (see Klein & Hilchey, 2011) is thought to make use of specific oculomotor regions such as the superior colliculus (SC; Wang et al., 2011, 2012). Therefore, it could be that oculomotor IOR is a ubiquitous process (cf. Hilchey et al., 2018) that can get masked (Hilchey et al., 2018; Schöpper et al., 2022b) but not fully overwritten; in contrast, IOR resulting from manual responses might not as strongly be affected by ubiquitous oculomotor orienting mechanisms (cf. Redden et al., 2021, for a recent discussion of differences between manual and saccadic IOR) – thus being at risk to be overwritten by retrieval. Yet, there are observations of location repetition costs or IOR in manual discrimination tasks of arrow pointing direction with a long interval between stimuli (Avery et al., 2015; Ding et al., 2016; Weger et al., 2008). This could suggest that stimuli with attentional-shifting properties such as arrows (Pratt & Hommel, 2003; Pratt et al., 2010) might fuel orienting processes. However, if the discrimination of nonspatial target identity is based on processing targets without any spatial or attention-shifting properties like, for example, shapes (other than arrows) or colors, retrieval overwrites orienting effects in manual responding.

Our interpretation of the results hinges on the assumption that decay rates of IOR set in later than those for retrieval-based effects. However, it has been found that IOR is also reduced over time (e.g., Hu et al., 2011; Lupiáñez et al., 1997; see also Samuel & Kat, 2003). Thus, it is possible that IOR decays as quick as retrieval-based effects and the absence of IOR at later RSIs is simply caused by that. However, this would not explain the location repetition costs found for long intervals in eye movements (Schöpper et al., 2022b) and arrow discrimination tasks (Avery et al., 2015; Ding et al., 2016; Weger et al., 2008).

Next to explanations based on processes that involve, for example, inhibition (Klein, 2000; Posner & Cohen, 1984), IOR has also been explained by a detection cost (Lupiáñez, 2010; Lupiáñez et al., 2013): A response to a target that repeats its location is slowed because the target is “absorbed” by the activation of the previously presented stimulus. Crucially, the previous stimulus is thought to be integrated into an object file (Kahneman et al., 1992) and the newly appearing target sharing the same location is perceived as belonging to the latter. As the detection cost theory refers to object files just as theories on binding and retrieval do (see Hommel, 2004), IOR could be seen as a retrieval-based effect – and thus should be affected by the same decay rates as S-R binding. In line with this we found strongest IOR at the short RSIs which was reduced at longer RSIs – just as predicted by the detection cost theory, which proposes maximum interference of same location stimuli at short intervals between stimuli (Lupiáñez, 2010; Lupiáñez et al., 2013). With that in mind, the argument of long-lasting IOR at long intervals between responses (e.g., Avery et al., 2015; Schöpper et al., 2022b) might be seen as in contrast with this; however, it is possible that the decay rate is identical, but the strength of the initial effect differs (cf. Frings et al., 2022; Hommel & Frings, 2020; for a discussion, see Frings, Foerster, et al., 2024). In other words, in studies in which IOR is observed at long intervals between responses, the experimental design (e.g., due the involvement of the oculomotor system; Schöpper et al., 2022b) might simply produce large effects that – even when decaying at the same speed (Frings et al., 2022) – are still observed late in time (cf. Hommel & Frings, 2020; Moeller & Frings, 2021).

Of note, in the current study binding effects were still evident at the longest RSIs, likely due to response x location binding effects being quite strong (e.g., Hilchey et al., 2017; Schöpper & Frings, 2024; Schöpper et al., 2020). Thus, it is possible that even a minimum of retrieval blurs IOR.

## Conclusion

Discriminating non-spatial features of targets repeating or changing their locations leads to effects of binding and retrieval. In contrast, IOR is only rarely observed or at least reduced in such tasks. The current results show that the decreasing effect of retrieval on responding with increasing RSI does not lead to IOR gaining in size. This suggests that retrieval-based processes do not simply mask (Hilchey et al., 2018; Klein, 2004; Schöpper et al., 2022b) but rather overwrite IOR.

## Data Accessibility Statement

Data of the experiments is available at https://doi.org/10.23668/psycharchives.15927. Code for analysis is available at https://doi.org/10.23668/psycharchives.15928.

## Footnotes

Gabay et al. (2012) had a discrimination task in which they instructed participants to remember the cue identity and execute a yes/no response (e.g., “Was the cue an X?”) after target response execution using the same keys. However, in the current study all responses are executed based on perceptual features and do not involve a memory task.

However, see Geissler et al. (2024), Hilchey et al. (2020), and Schöpper et al. (2022a) for binding effects in detection and localization performance when post-selective processing of the location feature is necessary.

Note that responding to central arrow targets does not necessarily produce IOR (e.g., Schöpper & Frings, 2022; Taylor & Klein, 2000). More general, although several experimental setups lead to location repetition costs, there is a debate if all of these measure the same concept of IOR (e.g., Dukewich & Klein, 2015; Hilchey et al., 2014). However, we mention these in this context, as they are discussed as inhibitory processes.

Note that in the design as used in the current study, color and response are completely confounded (as, for example, in Schöpper et al., 2020, and Schöpper & Frings, 2024) and that a binding effect can be caused by binding between location and color, location and response, or both. However, color and location usually do not bind together (Hommel, 1998, 2007), suggesting that the binding effect will mostly be caused by binding between location and response (Hilchey et al., 2017).

It has been found that the location of a stimulus can have an impact on the strength of IOR (Soballa et al., 2022; Spalek & Hammad, 2004) or that there are at least overall reaction time differences dependent on target location (Snyder & Schmidt, 2014). We thus added probe target location (bottom vs. top) as a factor to the analysis. Participants were faster for top (503 ms) compared to bottom targets (508 ms), *F*(1, 29) = 9.26, *p* = .005, \[
\eta_p^2
\] = .24. This effect was modulated by RSI, *F*(5, 25) = 2.72, *p* = .043, \[
\eta_p^2
\] = .35 (Bottom: 500: 510 ms; 1000: 499 ms; 1500: 505 ms; 2000: 512 ms; 2500: 508 ms; 3000: 515 ms; Top: 500: 505 ms; 1000: 496 ms; 1500: 502 ms; 2000: 507 ms; 2500: 507 ms; 3000: 501 ms). However, position did not modulate the effects of interest, that is, response relation, location relation, or the interaction of the latter (all *F* ≤ 0.60, all *p* ≥ .446); the remaining higher order interactions were also unmodulated by position (all *F* ≤ 1.67, all *p* ≥ .178).

There was a tendency for the interaction of location relation, RSI, and position which, however, did not reach significance, *F*(5, 25) = 2.19, *p* = .087, \[
\eta_p^2
\] = .31. There was no other impact of position (all *F* ≤ 0.32, all *p* ≥ .578).

Participants were faster for top (471 ms) compared to bottom targets (474 ms), *F*(1, 29) = 8.37, *p* = .007, \[
\eta_p^2
\] = .22. Position did not modulate any effect or interaction (all *F* ≤ 1.37, all *p* ≥ .252).

There was a tendency for position modulating location relation, *F*(1, 29) = 3.82, *p* = .060, \[
\eta_p^2
\] = .12, further being modulated by RSI, *F*(5, 25) = 2.22, *p* = .084, \[
\eta_p^2
\] = .31; yet, these did not reach statistical significance. There was no other impact of position (all *F* ≤ 1.99, all *p* ≥ .116).

## Appendix A1

t-tests against zero for each effect of interest in reaction times, separate for each RSI and experiment. S-R Binding = Stimulus-response binding effect, IOR = Inhibition of return, RRB = Response repetition benefit.

**Table:**

EXPERIMENT	EFFECT	RSI	*M*	*SD*	*t*(1, 29)	*p*	*d*

**Experiment 1**	S-R Binding	500	52	40	7.04	<.001	1.29

		1000	45	40	6.21	<.001	1.13

		1500	39	41	5.21	<.001	0.95

		2000	20	40	2.80	.009	0.51

		2500	30	36	4.45	<.001	0.81

		3000	14	54	1.39	<.001	0.25

	IOR	500	–5	20	–1.49	.147	0.27

		1000	–1	18	–0.31	.760	0.06

		1500	–2	28	–0.36	.720	0.07

		2000	–5	30	–0.92	.364	0.17

		2500	2	24	0.42	.679	0.08

		3000	2	22	0.38	.705	0.07

	RRB	500	52	34	8.21	<.001	1.50

		1000	37	30	6.79	<.001	1.24

		1500	30	28	5.94	<.001	1.08

		2000	21	27	4.36	<.001	0.80

		2500	17	29	3.15	.004	0.57

		3000	11	30	1.90	.067	0.35

**Experiment 2**	S-R Binding	500	85	45	10.35	<.001	1.89

		1000	62	33	10.20	<.001	1.86

		1500	48	49	5.36	<.001	0.98

		2000	30	41	3.99	<.001	0.73

		2500	45	34	7.10	<.001	1.30

		3000	27	33	4.50	<.001	0.82

	IOR	500	–4	26	–0.78	.441	0.14

		1000	–9	23	–2.23	.034	0.41

		1500	–3	17	–1.08	.289	0.20

		2000	–8	14	–3.23	.003	0.59

		2500	0	21	–0.11	.914	0.02

		3000	3	17	1.02	.318	0.19

	RRB	500	31	26	6.52	<.001	1.19

		1000	27	28	5.21	<.001	0.95

		1500	25	31	4.51	<.001	0.82

		2000	10	28	2.02	.052	0.37

		2500	15	24	3.49	.002	0.64

		3000	5	25	1.20	.240	0.22

## Appendix A2

t-tests against zero for each effect of interest in error rates, separate for each RSI and experiment. S-R Binding = Stimulus-response binding effect, IOR = Inhibition of return, RRB = Response repetition benefit.

**Table:**

EXPERIMENT	EFFECT	RSI	*M*	*SD*	*t*(1, 29)	*p*	*d*

**Experiment 1**	S-R Binding	500	5.91	7.90	4.10	<.001	0.75

		1000	6.21	8.54	3.98	<.001	0.73

		1500	3.77	8.33	2.48	.019	0.45

		2000	4.82	8.57	3.08	.005	0.56

		2500	2.37	8.59	1.51	.141	0.28

		3000	4.23	9.14	2.53	.017	0.46

	IOR	500	–0.76	2.88	–1.45	.158	0.27

		1000	–1.87	3.61	–2.84	.008	0.52

		1500	–0.23	2.89	–0.43	.669	0.08

		2000	1.23	4.16	1.62	.116	0.30

		2500	0.96	4.95	1.06	.298	0.19

		3000	0.01	4.05	0.01	.989	0.00

	RRB	500	2.27	3.10	4.02	<.001	0.73

		1000	2.10	4.28	2.69	.012	0.49

		1500	0.26	3.17	0.44	.661	0.08

		2000	–0.40	4.35	–0.51	.618	0.09

		2500	–0.53	3.56	–0.81	.426	0.15

		3000	0.21	4.08	0.29	.777	0.05

**Experiment 2**	S-R Binding	500	11.91	6.52	10.01	<.001	1.83

		1000	8.64	9.77	4.84	<.001	0.88

		1500	5.07	6.14	4.52	<.001	0.83

		2000	3.85	8.13	2.60	.015	0.47

		2500	2.33	6.77	1.89	.069	0.35

		3000	3.22	8.75	2.01	.053	0.37

	IOR	500	–1.48	4.55	–1.78	.086	0.33

		1000	–0.36	3.54	–0.56	.582	0.10

		1500	0.38	3.59	0.59	.562	0.11

		2000	0.59	3.55	0.91	.373	0.17

		2500	1.32	4.63	1.56	.129	0.29

		3000	–0.52	3.61	–0.80	.432	0.15

	RRB	500	2.08	4.31	2.65	.013	0.48

		1000	1.19	3.34	1.95	.061	0.36

		1500	0.43	5.23	0.45	.653	0.08

		2000	–0.70	3.66	–1.04	.307	0.19

		2500	0.33	3.96	0.46	.648	0.08

		3000	0.13	5.47	0.13	.897	0.02

## Appendix A3

We performed a 2 (Experiment: Trialwise vs. Blockwise) × 6 (RSI: 500 × 1000 × 1500 × 2000 × 2500 × 3000 ms) × 2 (response relation: repetition vs. change) × 2 (location relation: repetition vs. change) repeated-measures MANOVA on probe reaction times. The main effect of experiment was significant, *F*(1, 58) = 4.95, *p* = .030, \[
\eta_p^2
\] = .08, with participants responding slower in Experiment 1 (506 ms) compared to Experiment 2 (473 ms). The main effect of RSI was significant, *F*(5, 54) = 16.32, *p* < .001, \[
\eta_p^2
\] = .60 (500: 471 ms; 1000: 472 ms; 1500: 488 ms; 2000: 499 ms; 2500: 500 ms; 3000: 504 ms), and further modulated by experiment, *F*(5, 54) = 12.56, *p* < .001, \[
\eta_p^2
\] = .54, showing that in Experiment 2 responses became slower with increasing RSIs (500: 435 ms; 1000: 446 ms; 1500: 473 ms; 2000: 488 ms; 2500: 492 ms; 3000: 501 ms) which was not observed in Experiment 1 (500: 508 ms; 1000: 498 ms; 1500: 503 ms; 2000: 510 ms; 2500: 507 ms; 3000: 508 ms). The main effect of response relation was significant, *F*(1, 58) = 68.23, *p* < .001, \[
\eta_p^2
\] = .54 (RR: 477 ms; RC: 501 ms) and was further modulated by RSI, *F*(5, 54) = 26.25, *p* < .001, \[
\eta_p^2
\] = .71 (500 [RR: 451 ms; RC: 492 ms]; 1000 [RR: 456 ms; RC: 488 ms]; 1500 [RR: 474 ms; RC: 502 ms]; 2000 [RR: 491 ms; RC: 507 ms]; 2500 [RR: 492 ms; RC: 508 ms]; 3000 [RR: 501 ms; RC: 508 ms]), but not by experiment *F*(1, 58) = 2.39, *p* = .128, \[
\eta_p^2
\] = .04. The main effect of location relation was significant, *F*(1, 58) = 4.30, *p* = .043, \[
\eta_p^2
\] = .07, and depicted IOR (LR: 490 ms; LC: 488 ms), but was not modulated by RSI, *F*(5, 54) = 1.79, *p* = .131, \[
\eta_p^2
\] = .14, or experiment *F*(1, 58) = 0.58, *p* = .448, \[
\eta_p^2
\] = .01. The interaction of response relation and location relation was significant, *F*(1, 58) = 220.90, *p* < .001, \[
\eta_p^2
\] = .79 (RRLR: 468 ms; RRLC: 486 ms; RCLR: 513 ms; RCLC: 489 ms), and further modulated by experiment, *F*(1, 58) = 8.43, *p* = .005, \[
\eta_p^2
\] = .13, and RSI, *F*(5, 54) = 11.51, *p* < .001, \[
\eta_p^2
\] = .52. All remaining interactions involving the experimental factor were not significant (all *F* ≤ 1.58; all *p* ≥ .180).

## Appendix A4

We performed the same 2 (Experiment: Trialwise vs. Blockwise) × 6 (RSI: 500 × 1000 × 1500 × 2000 × 2500 × 3000 ms) × 2 (response relation: repetition vs. change) × 2 (location relation: repetition vs. change) repeated-measures MANOVA on probe error rates. The main effects of experiment, *F*(1, 58) = 0.01, *p* = .941, \[
\eta_p^2
\] = .00, RSI, *F*(5, 54) = 2.21, *p* = .066, \[
\eta_p^2
\] = .17, and location, *F*(1, 58) = 0.09, *p* = .760, \[
\eta_p^2
\] < .01, were not significant. The main effect of response relation was significant, *F*(1, 58) = 4.79, *p* = .033, \[
\eta_p^2
\] = .08 (RR: 3.48%; RC: 4.10%) and was further modulated by RSI, *F*(5, 54) = 4.12, *p* = .003, \[
\eta_p^2
\] = .28 (500 [RR: 2.18%; RC: 4.36%]; 1000 [RR: 2.75%; RC: 4.40%]; 1500 [RR: 3.21%; RC: 3.55%]; 2000 [RR: 4.16%; RC: 3.62%]; 2500 [RR: 4.23%; RC: 4.14%]; 3000 [RR: 4.36%; RC: 4.53%]). Location relation and RSI interacted, *F*(5, 54) = 3.60, *p* = .007, \[
\eta_p^2
\] = .25. The interaction of response relation and location relation was significant, *F*(1, 58) = 65.86, *p* < .001, \[
\eta_p^2
\] = .53 (RRLR: 2.21%; RRLC: 4.75%; RCLR: 5.43%; RCLC: 2.77%), and was further modulated by RSI, *F*(5, 54) = 6.39, *p* < .001, \[
\eta_p^2
\] = .37. All remaining interactions involving the experimental factor were not significant (all *F* ≤ 2.03; all *p* ≥ .090).
